# RAG1 deficiency durably alters dermal group 2 innate lymphoid cells and modifies contact hypersensitivity

**DOI:** 10.3389/fimmu.2026.1859649

**Published:** 2026-07-20

**Authors:** Mohamed M. Saleh, Kexin Liao, Andrea Braun, Gabriela Salinas, Michael P. Schön, Prasad Dasari, Timo Buhl

**Affiliations:** 1 Department of Dermatology, Venereology, and Allergology, University Medical Center, Georg August University, Göttingen, Germany; 2 Department of Neurology, University Hospital of Bonn, Bonn, Germany; 3 NGS- Integrative Genomics Core Unit, Institute of Pathology, University Medical Center Göttingen, Göttingen, Germany; 4 Lower Saxony Institute of Occupational Dermatology, University Medical Center Göttingen, Göttingen, Germany

**Keywords:** contact hypersensitivity, cutaneous immunity, dermal group 2 innate lymphoid cells, IL-7 receptor, innate lymphoid cells, RAG1 deficiency, skin inflammation

## Abstract

**Introduction:**

Dermal group 2 innate lymphoid cells (dILC2s) contribute to skin homeostasis and inflammatory responses, yet they are frequently studied in Rag1-deficient mice in which the dILC2 compartment may itself be altered.

**Methods:**

We compared dILC2s from wild-type (WT) and Rag1-deficient mice by transcriptomic profiling, flow cytometric phenotyping, and functional analysis in a DNFB-induced contact hypersensitivity model with short-term adoptive lymphocyte transfer.

**Results:**

Rag1 deficiency was associated with a marked expansion of dILC2s and broad transcriptional remodeling, including increased expression of Il7r, Thy1, Il5, and Il13, together with enrichment of cytokine signaling, apoptosis, and activation pathways. *In vivo*, Rag1-deficient mice showed attenuated ear swelling after DNFB challenge and, in contrast to WT mice, failed to expand their absolute dILC2 abundance during inflammation. Short-term adoptive transfer of sensitized WT lymphocytes modestly modified dILC2 dynamics but did not restore the WT inflammatory phenotype. At the cellular level, Rag1-deficient dILC2s displayed a predominantly CD44hi phenotype, increased basal apoptosis, elevated caspase-3/7 activity, and increased BrdU incorporation.

**Discussion:**

Together, these findings show that Rag1 deficiency durably alters dILC2 state and responsiveness and should be taken into account when interpreting cutaneous inflammation models in immunodeficient mice.

## Introduction

Innate lymphoid cells (ILCs) reside in barrier tissues such as lung, gut, and skin, where they act as rapid sentinels to environmental stimuli. ILCs belong to the innate immune system, but functionally, they mirror the effector programs of adaptive lymphocytes (1, 2). Among them, group 2 ILCs (ILC2s) express GATA3 and ICOS and secrete type-2 cytokines, thereby contributing to tissue homeostasis, helminth defense, and allergic inflammation (1, 3). They rapidly produce cytokines such as IL-4, IL-5, and IL-13 in response to epithelial cell-derived signals (e.g., IL-33, TSLP, IL-25), which drive eosinophil recruitment, mast cell modulation and barrier integrity (4–6). ILC2s exhibit marked tissue specificity, and dermal ILC2s (dILC2s) form a distinct population characterized by high expression of the skin-homing receptor CD103 (integrin αE) (5, 7) and elevated IL-25R and KLRG1 expression relative to pulmonary ILC2s (8). Moreover, environmental cues may drive substantial ILC2 plasticity, enabling transitions across functional states and even other types of ILCs (3, 9). For instance, in the lung, natural and inflammatory ILC2 subsets (nILC2s and iILC2s, respectively) can be distinguished by CD44 and CD90 expression (10). Whether analogous phenotypes exist in the dILC2s remains unclear.

Skin ILC2s differ between mice and humans in abundance, phenotype, and tissue regulation. In mice, dermal ILC2s are abundant, constitutively produce IL-13, interact with mast cells, and depend on IL-7 for their maintenance (5). Human skin ILC2s appear more heterogeneous and are shaped by the local tissue environment, including microbiome and neuroimmune signals (3, 8).

dILC2s have been implicated in several inflammatory skin diseases, including psoriasis, atopic dermatitis, and contact hypersensitivity (CHS) (4, 6, 11–13) but their function appears to be context dependent. For instance, depletion of dILC2s exacerbates CHS inflammation suggesting a regulatory function (6), whereas in MC903-induced atopic dermatitis dILC2s amplify inflammation (14). These divergent findings underscore that dILC2 function is shaped by the local cytokine milieu and tissue state. Previous analyses of dILC2 involvement in CHS have primarily relied on TNCB-based models. The classical hapten DNFB, which induces a robust CHS response, has remained incompletely investigated in the context of dILC2 biology.

Studying dILC2s *in vivo* is technically challenging due to their low abundance. Rag1-deficient (*Rag1*^-^/^-^*)* mice, which lack mature T and B cells, exhibit a 2-3-fold increase in dILC2 numbers (5, 14), and therefore serve as a common model for ILC2 research. RAG1 is essential for V(D)J recombination, but accumulating evidence shows additional roles in genome stability and cellular fitness (14–16). Intriguingly, a subset of ILC2s expresses RAG1 during development, and loss of RAG activity has been linked to altered fitness and functional properties in innate lymphocyte populations (14, 16). Recent work demonstrated that developmental RAG1 acts as an immunological suppressor in dILC2s and that its absence results in a “hyperactivated phenotype” with enhanced skin inflammation in MC903-induced dermatitis (14). However, these studies compared RAG1-expressing and RAG1-naïve dILC2s within wild-type (WT) mice. The transcriptional and functional consequences of complete Rag1 deficiency for dILC2s in skin inflammation remain incompletely defined.

Therefore, an important unresolved aspect in cutaneous immunology is whether dILC2s from *Rag1^-^/^-^* mice differ from WT dILC2s in their transcriptional profile and differentiation, and how such differences influence cutaneous inflammatory responses. This issue is particularly relevant because *Rag1^-^/^-^* mice are widely used to interrogate ILC2 function, yet the absence of adaptive lymphocytes may alter the ILC2 compartment through durable changes in the host environment and loss of cellular crosstalk. Understanding how RAG1 deficiency affects dILC2 biology is essential for accurate interpretation of studies relying on *Rag1^-^/^-^* models.

In this study, we provide a skin-focused analysis of how Rag1 deficiency reshapes the dILC2 compartment under homeostatic and inflammatory conditions and how these changes relate to interactions with adaptive immune cells.

## Methods

### Mice

Female C57BL/6 mice were reared under specific pathogen-free conditions at the University of Göttingen Animal Facility. C57BL/6 WT, *Rag1*^-^/^-^, *Myd88*^-^/^-^, *Tlr3*^-^/^-^, and *Myd88xTlr3*^-^/^-^ mice were obtained from The Jackson Laboratory, Bar Harbor, ME. All mice used were 8–13 weeks old. All animal experiments were conducted in accordance with institutional and national regulations for animal welfare and were approved by the responsible local authorities.

### Contact hypersensitivity

Contact hypersensitivity (CHS) was induced as described previously (22). Briefly, mice were shaved on the back and sensitized on days 0 and 1 by topical application of 20 µl of 0.5% 1-chloro-2,4-dinitrobenzene (DNFB; Sigma Aldrich, Munich, Germany) dissolved in 4:1 acetone/olive oil (vehicle). On day 5, the right ear of the mice was challenged with 20 µl of 0.25% DNFB, while the left ear received 20 µl of vehicle only. After 24 hours DNFB-induced ear inflammation was assessed by measuring ear thickness in three different ear locations and averaging the results, using a thickness gauge C220T (Kroeplin, Schlüchtern, Germany). Mice were anesthetized with isoflurane (induction at 3–4%, maintenance at 1.5–2% in oxygen) and euthanized by CO_2_ inhalation followed by cervical dislocation. All procedures were performed in accordance with institutional and national animal welfare regulations.

### Adoptive transfer of lymphocytes

WT mice were shaved on the back and sensitized on day 0 and day 1 by DNFB. After five days, for adoptive transfer experiments, single-cell suspensions from auricular, cervical and inguinal lymph nodes of WT mice were prepared, and 2 × 10 (7) total lymphocytes were intravenously injected into *Rag1^-^/^-^* recipient mice. Cell viability was assessed before transfer by trypan blue exclusion. 24 hours later, control mice and adoptively-transferred mice were challenged with DNFB on the ears. Ear swellings and cellular infiltration were assessed 24 hours after challenge.

### Tissue processing and flow cytometry

Mouse ears were removed 24 hours after DNFB challenge, and the dorsal and ventral sides of the ear were separated. The ear tissue was minced into small pieces and digested with Liberase (Roche Diagnostics, Mannheim, Germany) and 0.1% DNase I (Roche Diagnostics, Mannheim, Germany) in 500 µl RPMI medium (Lonza, Verviers, Belgium) for 90 minutes at 37 °C. Released cells were filtered through a 50 µm filter (BD, Heidelberg, Germany) to obtain single-cell suspensions. Cells were counted and transferred to FACS tubes. After Fc-receptor blocking with TruStain fcX™ (BioLegend), the cells were stained with the following fluorochrome-conjugated antibodies: CD2 (RM2-5), CD3 (145-2C11), CD3 (17A2), CD11b (M1/70), CD25 (eBioPC.615), CD45 (30-F11), CD86 (GL-1), CD90 (53-2.1), CD103 (2E7), CD127 (SB/199); all from BioLegend, except of CD11b and CD25 (eBioScience) for 30 minutes at 4 °C. For negative controls, we prepared corresponding fluorescence-minus-one (FMO) probes for each fluorochrome and a single and unstained probe. Viability was determined with Zombie NIR (BioLegend). After washing, cells were analyzed using BD FACS Canto II (BD, Heidelberg, Germany). The gating strategy was designed to identify dILC2s within viable CD45^+^CD90hi cells while excluding CD2/CD3 signal-positive populations; FMO controls were used to define gating thresholds. Because CD2 and CD3 were acquired in a shared fluorescence channel in these experiments, CD2/CD3 signal-positive populations could not be further separated into CD2^+^ and CD3^+^ subsets.

### Annexin V staining, caspase 3/7 measurement, and BrdU assays

For Annexin V measurement, sensitization and challenge were performed as described above. This time, cells were harvested after 2 hours DNFB challenge, to detect early apoptotic events. The cells were then stained with Annexin V Staining Kit (BioLegend, Koblenz, Germany) following manufacturer’s instruction.

For detection of caspase 3/7 cleavage products, we used the Caspase 3/7 Staining Kit (ThermoFisher, Darmstadt, Germany). Sensitization, challenge and ear tissue preparation were performed as described above.

To determine dILC2 proliferation, 2 mg of BrdU were injected intraperitoneally at the time of DNFB challenge. After 24 hours, ear single-cell suspensions were prepared as described above and stained with BrdU Staining Kit according to the manufacturer’s protocol (BD, Heidelberg, Germany).

### *Ex vivo* stimulation

For *ex vivo* stimulation, ear tissue was processed into single-cell suspensions as described above. Cells were resuspended in complete RPMI medium and stimulated with phorbol 12-myristate 13-acetate (PMA; 50 ng/mL; Sigma-Aldrich) and ionomycin (1 µg/mL; Sigma-Aldrich). Samples were incubated for 6–16 hours at 37 °C in a humidified CO_2_ incubator. After incubation, cells were washed and prepared for flow cytometric analysis.

### RNA sequencing

RNA sequencing was performed on sorted dermal ILC2 isolated from pooled mice. For WT samples, cells from 30 mice were pooled into three independent biological replicates, each consisting of cells from 10 mice. For *Rag1^-^/^-^* samples, cells from 18 mice were pooled into two independent biological replicates, each consisting of cells from 9 mice, to obtain sufficient cell numbers for downstream analyses. Libraries were prepared using standard poly(A)-enrichment protocols and sequenced on an Illumina platform. Differential gene expression analysis was conducted using DESeq2 with genotype as the primary factor in the design formula. Quality control included principal component analysis and inspection of sample clustering. Subsequently, reads were counted for each gene using the Mus musculus reference genome (GRCm38) and annotation file Mus_musculus.GRCm38.84.gtf with featureCounts (v1.5.0-p1) (26). Read counts were analyzed in the R/Bioconductor environment using DESeq2 (27). Differentially expressed genes were defined *post hoc* using an absolute log2 fold-change >1 and an FDR-adjusted p-value <0.05. Gene annotation was performed using the Mus musculus dataset in biomaRt R package (28). Functional enrichment analyses were performed using the clusterProfiler R package, including Gene Ontology (GO) and Kyoto Encyclopedia of Genes and Genomes (KEGG) pathway analyses, as well as Gene Set Enrichment Analysis (GSEA) based on ranked gene lists (29, 30). For GSEA, all detected genes were ranked by log2 fold-change without prior filtering. Heatmaps were generated using the ComplexHeatmap R package to visualize enrichment and expression patterns (31). All analyses were conducted in R using curated GO and KEGG databases (32, 33).

### Statistical analysis

Statistical significance between two independent groups was assessed using either unpaired *t*-tests or Welch’s t-tests, as indicated in the respective figure legends. Paired two-tailed t-tests were used for comparisons of vehicle- and DNFB-treated ears from the same mice. For multiple comparisons, significance was determined by one-way ANOVA followed by a Dunnett’s multiple correction. For experiments involving two independent variables (genotype and treatment), two-way ANOVA followed by appropriate multiple-comparison correction was applied. A p-value of ≤ 0.05 was considered statistically significant. Statistical tests performed in each experiment are depicted in figure legends. *p≤ 0.05; **p≤ 0.01; ***p≤ 0.001 and ****p≤ 0.0001. Statistical analyses were conducted with GraphPad Prism version 10.3.

### Ethics statement

All animal experiments were approved by the responsible local authorities and conducted in accordance with institutional guidelines and national regulations for animal welfare. Mice were housed under specific pathogen–free conditions, and all procedures were performed to minimize animal suffering.

### Data availability

RNA sequencing data have been deposited in the NCBI Gene Expression Omnibus (GEO; accession number: GSE317014). All other data supporting the findings of this study are available from the corresponding authors upon reasonable request.

## Results

### Rag1 deficiency expands and transcriptionally remodels dermal group 2 innate lymphoid cells

**Figure 1:**
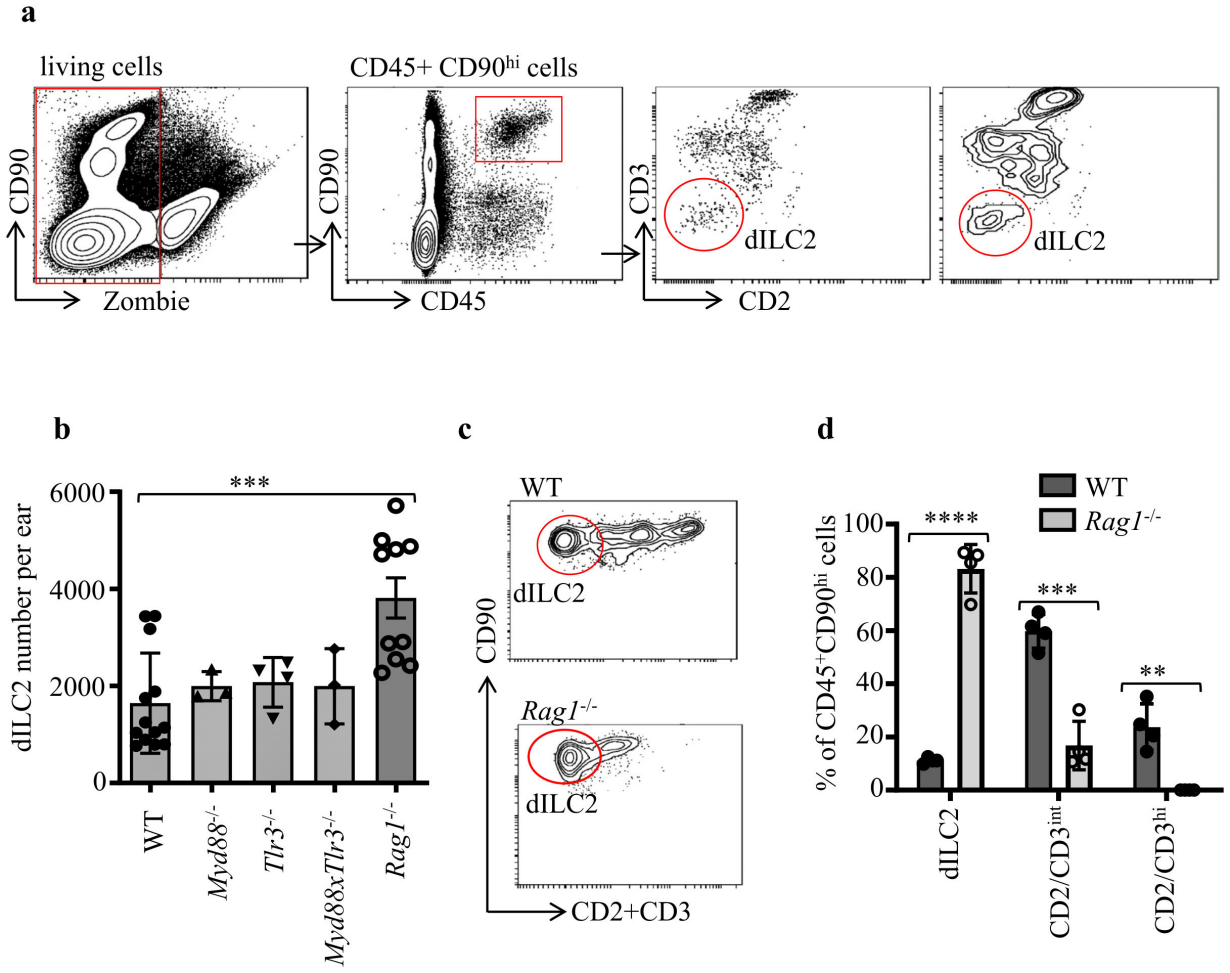
*Rag1*^-^/^-^ mice exhibit increased dILC2 numbers relative to C57BL/6 WT, *Myd88*^-^/^-^, *Tlr3*^-^/^-^, and *Myd88xTlr3*^-^/^-^ mice. **(A)** Exemplary gating strategy for identification of dermal ILC2s within viable CD45+CD90hi ear skin cells in WT/BL6 mice. The two right-most panels show the same CD2/CD3 versus CD90 gate as a conventional dot plot and as a density/contour plot, respectively. **(B)** Total dILC2 numbers of ear-skin of different mouse strains (C57BL/6 WT, *Rag1*^-^/^-^, *Myd88*^-^/^-^, *Tlr3*^-^/^-^, and *Myd88xTlr3*^-^/^-^), quantified by flow cytometry. Cell numbers were derived from dILC2/200k counted cells and extrapolated to the whole ear. **(C)** Representative plots of WT (upper panel) and *Rag1^-^/^-^* (lower panel) ear skin CD45^+^CD90^hi^ cells showing dILC2s and the indicated CD2/CD3 signal-defined populations. **(D)** Percentage of dILC2s and the indicated CD2/CD3 signal-defined populations among CD45+CD90^hi^ cells in WT and *Rag1^-^/^-^* mice. Panel **(B)** shows mean ± SEM of n=3 (*Myd88*^-^/^-^, and *Myd88xTlr3*^-^/^-^), n=4 (*Tlr3*^-^/^-^), n=10 (*Rag1*^-^/^-^) or n=13 (WT) mice per group, pooled from 1–3 independent experiments (including cell culture and CHS experiments). Panel **(D)** shows mean ± SEM of n=5 mice per group from one experiment. For Panel **(B)** statistics, one-way ANOVA followed by a Dunnett’s multiple correction were used. For Panel **(D)** statistics, multiple unpaired *t-*tests separately for each row were used.

To compare dermal ILC2 abundance across selected innate signaling-deficient strains and *Rag1^-^/^-^* mice, we first quantified dILC2s using a validated gating strategy (**Figure 1A**). We found that *Myd88*^-^/^-^, *Tlr3*^-^/^-^, and *Myd88xTlr3*^-^/^-^ mice exhibited dILC2 numbers comparable to WT controls (**Figure 1B**). In contrast, *Rag1^-^/^-^* mice showed a marked increase in total dILC2 numbers (3813 ± 416 vs. 1646 ± 286 cells per ear; p ≤ 0.0001) and a disproportionately expanded dILC2 fraction within the CD45^+^CD90^hi^ compartment (**Figures 1C, D**). Despite numerical expansion, expression of canonical dILC2 markers, including GATA3 and ICOS, remained unchanged between genotypes (**Supplementary Figure 1**), suggesting that steady-state differentiation is preserved.

**Figure 2:**
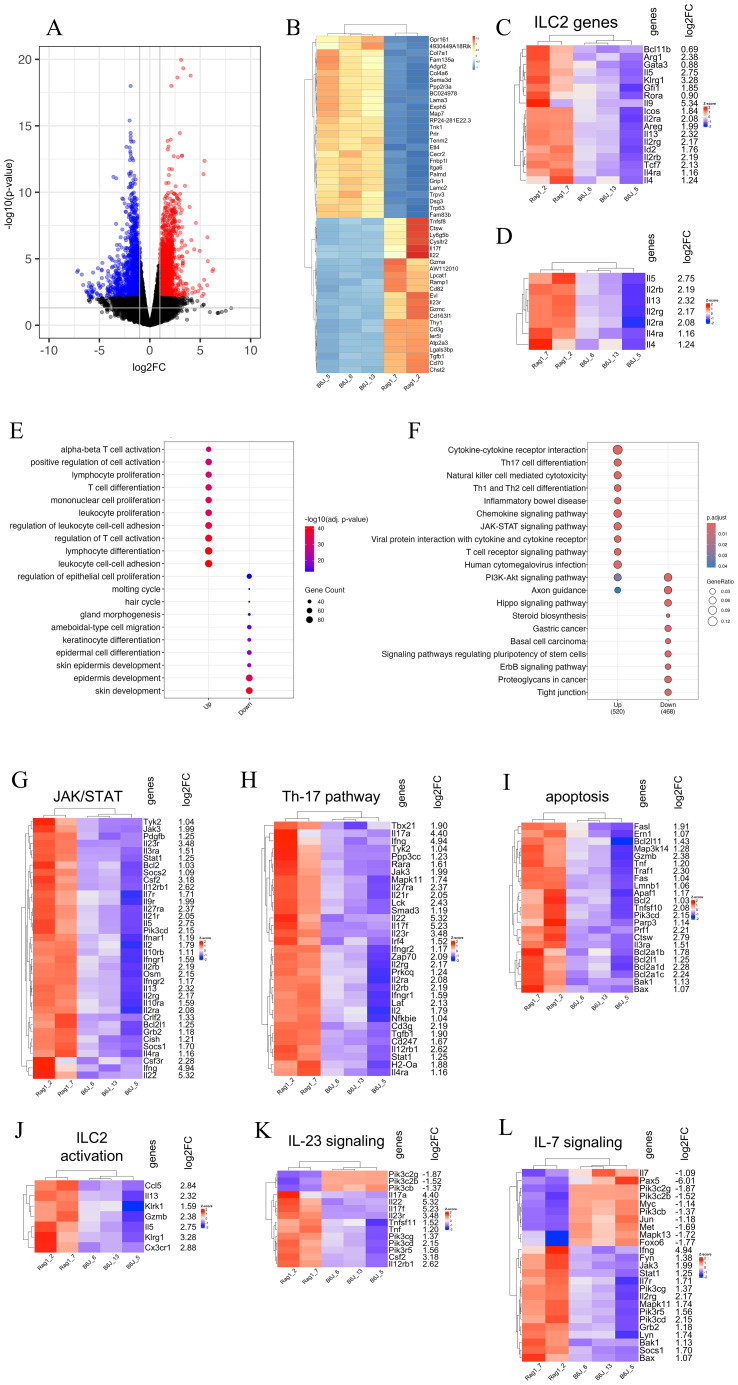
*Rag1*^-^/^-^ dILC2 show an altered gene expression profile compared to WT dILC2. **(A)** Volcano plot of differential gene expression in dILC2s between WT and *Rag1*^-^/^-^ mice. The volcano plot displays significantly upregulated genes in *Rag1*^-^/^-^ ILC2s in red, downregulated genes in blue, and non-significant genes in black compared to WT ILC2s. Significance was defined as an adjusted *p*-value < 0.05 and log_2_ fold change > 1. **(B)** Heat map of top 50 differentially regulated genes in dILC2s from WT and *Rag1*^-^/^-^ mice. **(C)** Heat map of ILC2-associated gene set and **(D)** ILC2-related cytokine gene set. **(E)** Bubble plot represents top ten enriched up and down regulated biological processes and **(F)** KEGG pathways for upregulated and downregulated genes, ranked by adjusted *p*-value. Heat map of enriched gene sets of **(G)** JAK/Stat signalling pathway, **(H)** Th-17 signalling, **(I)** apoptosis pathway, **(J)** ILC activation, **(K)** IL-23 signalling, and **(L)** IL-7 signalling. Only the most significantly regulated genes/pathways are shown for clarity.

To determine whether RAG1 deficiency affects dILC2 identity beyond cell abundance, we performed RNA sequencing of sorted dILC2s. Differential expression analysis identified 2,503 significantly regulated genes, indicating extensive transcriptional remodeling (**Figures 2A, B**). Many ILC2-associated genes (*Il5, Il13, Il2ra, Il4ra*) were upregulated in *Rag1^-^/^-^* cells (**Figures 2C, D**). Pathway analyses revealed enrichment of cytokine signaling, proliferation, JAK/STAT and PI3K/Akt pathways, as well as IL-17–related programs, apoptosis and ILC activation (**Figures 2E–J**), consistent with altered activation thresholds and increased plasticity. Canonical IL-7 and IL-23 signaling pathways were enriched (**Figures 2K, L**).

Given the prominent upregulation of *Il7r* and *Thy1*, we validated protein expression by flow cytometry. *Rag1*^-^/^-^ dILC2s displayed significantly higher IL-7Rα and Thy1 levels compared with WT dILC2s (**Supplementary Figure 2**), whereas other transcriptionally elevated markers, such as ICOS or cytokines, showed no corresponding steady-state protein differences (**Supplementary Figure 1**). Collectively, these results demonstrate that RAG1 deficiency selectively expands the dILC2 compartment while inducing broad transcriptional alteration that affects key pathways of survival, activation, and lineage flexibility.

### Rag1-deficient mice show attenuated DNFB-induced contact hypersensitivity and aberrant dILC2 dynamics

**Figure 3:**
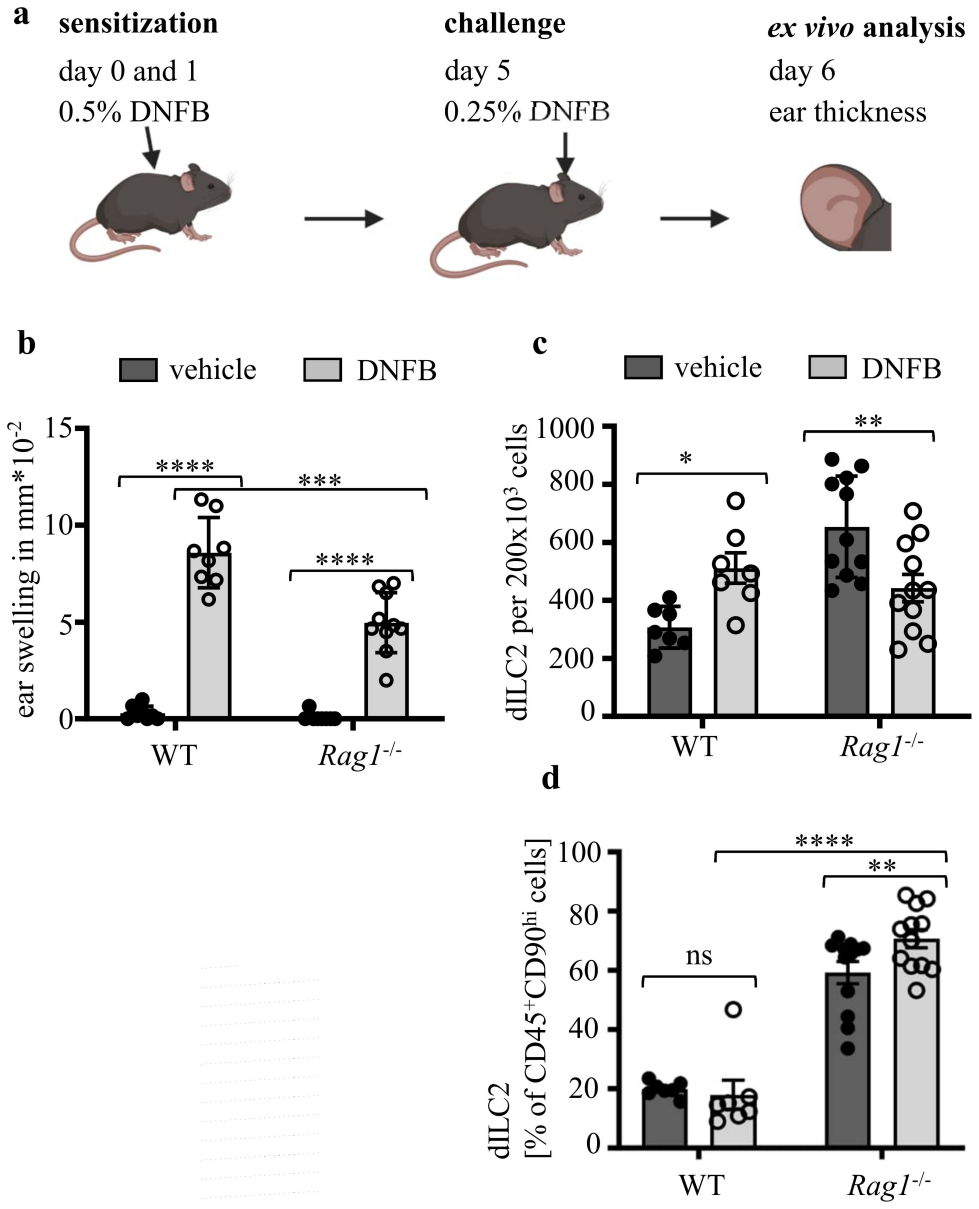
*Rag1^-^/^-^* mice show attenuated DNFB-induced contact hypersensitivity and altered dermal ILC2 dynamics. **(A)** Schematic representation of study design of CHS induction with DNFB, created with BioRender. Mice were sensitized with DNFB (0.5%) on days 0 and 1. On day 5, mouse ears were challenged with either DNFB (0.25%) or vehicle control. After 24h (day 6), DNFB-induced ear inflammation was determined by measuring the ear thickness. **(B)** Ear thickness in vehicle and DNFB-treated ears in WT and *Rag1*^-^/^-^ mice. **(C)** Number of dILC2 in DNFB and vehicle treated ears of WT and *Rag1*^-^/^-^ mice, per 200,000 measured cells. **(D)** Frequency of dILC2s among CD45^+^CD90hi cells in vehicle- and DNFB-treated ears of WT and *Rag1^-^/^-^* mice. Panels **(B–D)** show mean ± SEM of n=7 (WT) or n=11 (*Rag1*^-^/^-^) mice, respectively, of > 3 independent experiments. For vehicle-versus-DNFB comparisons within the same genotype, paired two-tailed t-tests were used. For comparisons between genotypes, unpaired two-tailed t-tests were used.

Given the extensive transcriptional alteration observed in *Rag1*^-^/^-^ dILC2s, we next examined their functional responses *in vivo* using a DNFB-induced CHS model (**Figure 3A**). Twenty-four hours after challenge, WT mice developed robust ear swelling, whereas *Rag1*^-^/^-^ mice displayed significantly reduced inflammation (8.6 ± 0.5 vs. 5.0 ± 0.64 × 10^-^² mm; p = 0.0003) (**Figure 3B**). We then assessed dILC2 numbers in challenged and vehicle-treated ears. In WT mice, DNFB treatment induced a strong increase in dILC2 abundance compared with vehicle controls (512 ± 56 vs. 307 ± 27; p = 0.0186). Strikingly, the opposite pattern was observed in Rag1^-^/^-^ mice: dILC2 numbers decreased following DNFB challenge (442 ± 48 vs. 654 ± 53; p = 0.0058) (**Figure 3C**). To further contextualize these divergent dynamics within the altered lymphoid compartment of *Rag1^-^/^-^* mice, we additionally quantified dILC2s as a fraction of CD45^+^CD90hi cells. In WT mice, DNFB challenge did not significantly change the relative dILC2 fraction within this compartment, whereas *Rag1^-^/^-^* mice displayed a markedly higher dILC2 fraction at baseline that further increased after DNFB challenge (**Figure 3D**). Thus, the altered dILC2 response in *Rag1^-^/^-^* mice was evident both in cell counts and in the relative composition of the CD45^+^CD90hi compartment.

### Short-term adoptive lymphocyte transfer modifies CD45^+^CD90hi compartment dynamics without restoring WT-like inflammation in Rag1-deficient mice

**Figure 4:**
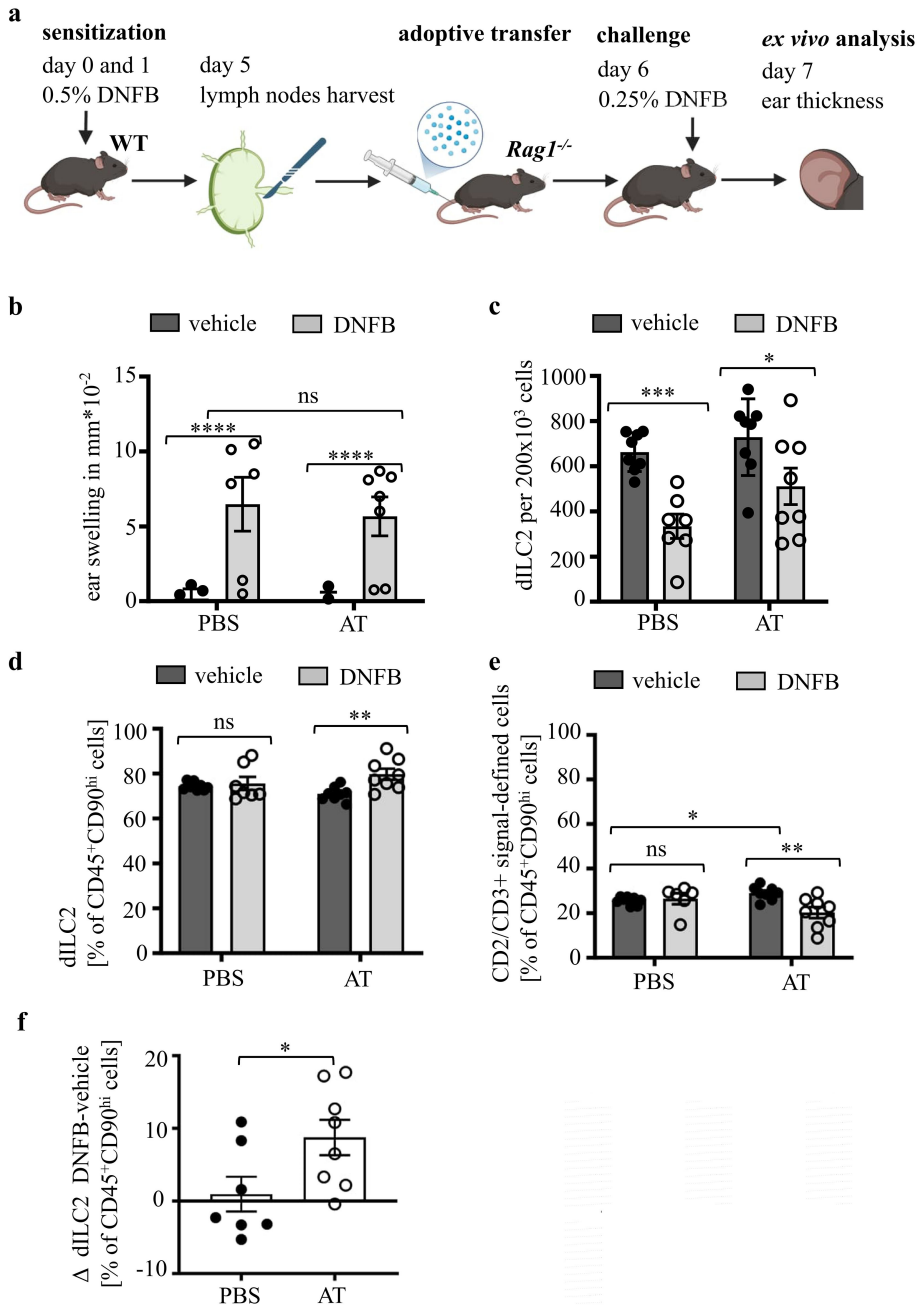
Short-term adoptive transfer of sensitized WT lymphocytes does not restore WT-like DNFB-induced inflammation but modifies the CD45^+^CD90hi lymphoid compartment in *Rag1^-^/^-^* mice. **(A)** Schematic representation of study design of adoptive transfer (AT) of lymphocytes into *Rag1*^-^/^-^ mice, created with BioRender. First, WT donor mice were sensitized with DNFB on days 0 and 1, as described previously. On day 5, lymphocytes were isolated from auricular, cervical and inguinal lymph nodes. Then, lymphocytes (2 × 10^7^)) were injected intravenously into non-sensitized *Rag1*^-^/^-^ recipient mice. 24 hours later, the ears were challenged with either DNFB or vehicle control. After 24h (corresponding to day 7), respectively, DNFB-induced ear inflammation was evaluated by measuring ear thickness and cellular analysis. **(B)** Ear thickness in vehicle and DNFB-treated ears in PBS mice (control) and AT mice. **(C)** Number of dILC2 in DNFB and vehicle treated ears of PBS- and AT-treated *Rag1*^-^/^-^ mice, per 200,000 measured cells. **(D)** Frequency of dILC2s among CD45^+^CD90hi cells in vehicle- and DNFB-treated ears of PBS- and AT-treated *Rag1^-^/^-^* mice. **(E)** Frequency of CD2/CD3 signal-defined cells among CD45^+^CD90hi cells in vehicle- and DNFB-treated ears of PBS- and AT-treated *Rag1^-^/^-^* mice. CD2 and CD3 were detected in a shared fluorescence channel; therefore, this analysis quantifies CD2/CD3 signal-defined cells and does not distinguish CD3^+^ T cells from CD2^+^ innate lymphoid or NK-lineage cells. **(F)** DNFB-induced change in the dILC2 fraction among CD45^+^CD90hi cells, calculated for each mouse as DNFB-treated ear minus vehicle-treated ear. Panels B–F show mean ± SEM of n = 7–8 mice per group, pooled from >3 independent experiments. Vehicle-versus-DNFB comparisons within the same experimental group were analyzed using paired two-tailed t-tests. Δ values in Panel F were compared between PBS- and AT-treated mice using an unpaired two-tailed Welch’s t-test.

We therefore explored whether short-term adoptive transfer (AT) of sensitized WT lymphocytes could modify these altered dILC2 dynamics during the elicitation phase. We performed AT of lymph node cells from DNFB-sensitized WT donors into naïve *Rag1*^-^/^-^ recipients (**Figure 4A**). Control *Rag1*^-^/^-^ mice received PBS. Following DNFB challenge, ear swelling remained comparable between AT and PBS groups (**Figure 4B**), indicating that short-term adoptive lymphocyte transfer does not restore the inflammatory response to WT levels. As expected, PBS-treated *Rag1*^-^/^-^ mice exhibited ear swelling consistent with the known irritant component of DNFB.

We next assessed dILC2 numbers in vehicle- and DNFB-treated ears. In PBS-treated *Rag1*^-^/^-^ controls, DNFB challenge reduced dILC2 abundance, mirroring findings from **Figure 3**. Adoptive lymphocyte transfer resulted in a trend toward higher dILC2 numbers after DNFB challenge compared with PBS-treated *Rag1^-^/^-^* controls, but this difference did not reach statistical significance (511 ± 81 vs. 334 ± 54; p = 0.101), and the WT expansion pattern was not restored (**Figure 4C**).

To determine whether short-term AT nevertheless altered the composition of the CD45^+^CD90hi compartment, we reanalyzed dILC2 frequencies within this gate. In PBS-treated *Rag1^-^/^-^* mice, DNFB did not significantly change the dILC2 fraction among CD45^+^CD90hi cells, whereas AT-treated *Rag1^-^/^-^* mice showed a significant DNFB-induced increase in this fraction (**Figure 4D**). Direct comparison of the paired DNFB–vehicle changes further showed that the DNFB-induced shift toward dILC2s was greater in AT-treated mice than in PBS-treated controls (**Figure 4F**). This shift was accompanied by a reciprocal decrease in CD2/CD3 signal-defined cells among CD45^+^CD90hi cells after DNFB challenge in AT-treated mice (**Figure 4E**). Because CD2 and CD3 were detected in a shared fluorescence channel, this analysis cannot distinguish transferred CD3^+^ T cells from endogenous CD2^+^ innate lymphoid or NK-lineage cells. Nevertheless, these data indicate that short-term AT measurably altered the DNFB-induced composition of the CD45^+^CD90hi lymphoid compartment, although it did not restore WT-like ear swelling or the absolute dILC2 expansion observed in WT mice.

### Rag1-deficient dILC2s display an activated, apoptosis-prone, proliferative phenotype

**Figure 5:**
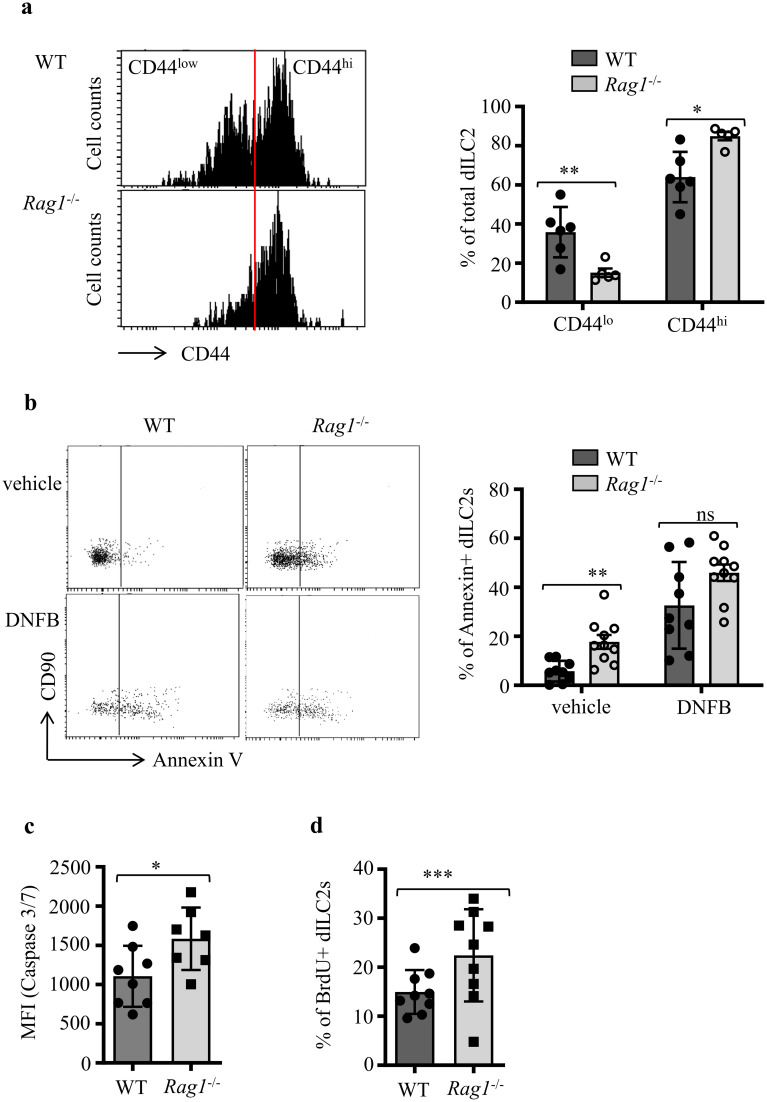
*Rag1^-^/^-^* dILC2s display an activated, apoptosis-prone, and proliferative phenotype. **(A)** Representative flow cytometry plots and corresponding quantification of CD44 expression in dILC2 of WT (upper gate) or *Rag1*^-^/^-^ (bottom gate) mice in DNFB treated ears after PMA/ionomycin activation. **(B)** Flow cytometry plots and corresponding quantification of Annexin V positive dILC2 in WT and *Rag1*^-^/^-^ mice in vehicle and DNFB-treated ears. **(C)** MFI of caspase 3/7 cleavage products of dILC2s from WT and *Rag1*^-^/^-^. **(D)** Percentage of BrdU positive dILC2 from WT and *Rag1*^-^/^-^ mice. For **(A)**, n=5 (*Rag1*^-^/^-^) or n=6 (WT) mice from two independent experiments were used. For **(B)**, n=9 (WT) or n=10 (*Rag1*^-^/^-^) of 3 independent experiments were used. For **(C)**, n=7–8 mice of three independent experiments were used. For **(D)** n= 7 (*Rag1*^-^/^-^) or n=9 (WT) mice of three independent experiments were used. For statistics, unpaired *t*-tests were used.

Given the impaired dILC2 expansion during CHS in *Rag1*^-^/^-^ mice, we next examined activation and stress responses at the single-cell level. Following DNFB challenge and ex vivo PMA/ionomycin stimulation, WT dILC2s segregated into CD44^lo^ and CD44^hi^ subsets, consistent with previously described (10) nILC2- and iILC2-like states (**Figure 5A**). In contrast, Rag1^-^/^-^ dILC2s predominantly exhibited a CD44^hi^ phenotype, indicating a shift toward an inflammatory activation state.

We then assessed apoptotic susceptibility. Early apoptosis, measured by Annexin V staining two hours after DNFB exposure, was increased in both genotypes; however, vehicle-treated *Rag1*^-^/^-^ dILC2s showed significantly higher baseline apoptosis compared with WT (18% ± 9 vs. 5.7% ± 4; p = 0.002) (**Figure 5B**). Consistently, *Rag1*^-^/^-^ dILC2s displayed elevated caspase-3/7 cleavage products (**Figure 5C**; **Supplementary Figure 3A**), indicating heightened stress sensitivity under steady-state conditions. Finally, *in vivo* proliferation was assessed by bromodeoxyuridine (BrdU) incorporation during CHS. *Rag1*^-^/^-^ dILC2s incorporated significantly more BrdU than WT cells (22.4% ± 3.1 vs. 14.9% ± 1.4; p = 0.047) (**Figure 5D**), demonstrating increased proliferative activity.

Together, these findings show that Rag1 deficiency is associated with a hyperactivated yet apoptosis-prone dILC2 phenotype with enhanced basal proliferation, providing a plausible cellular explanation for their altered behavior during cutaneous inflammation.

## Discussion

Group 2 innate lymphoid cells contribute to cutaneous immune homeostasis and type 2 immune responses, yet the mechanisms governing their development and functional diversification remain incompletely understood. Previous reports demonstrated that a subset of dILC2s expresses RAG1 during development and that RAG1-experienced cells differ from RAG1-naïve subsets (14, 16). In addition, RAG proteins have functions beyond V(D)J recombination, influencing genome integrity and cell survival (15, 16). Our findings extend these observations by showing that complete RAG1 deficiency results not only in increased dILC2 abundance but also in substantial transcriptional and functional alteration. These findings provide a skin-focused analysis of how Rag1 deficiency reshapes the dermal ILC2 compartment under homeostatic and inflammatory conditions and help explain why *Rag1^-^/^-^* mice display a distinct dILC2 state compared with WT mice.

We first confirmed earlier reports that *Rag1*^-^/^-^ mice exhibit markedly elevated numbers of dILC2s compared with WT controls (5, 11, 14). RNA sequencing revealed broad transcriptional alterations in *Rag1*^-^/^-^ dILC2s, with 1,209 genes upregulated and 1,294 downregulated relative to WT dILC2s. Several pathways associated with cytokine signaling, proliferation, plasticity, and apoptosis were enriched, consistent with previously reported roles of RAG1 in regulating lymphocyte fitness and genomic stability (15). Although RNA-seq does not always correlate with protein abundance, we validated increased expression of IL-7Rα and Thy1 – two key regulators of ILC2 survival and activation – on *Rag1*^-^/^-^ dILC2s. These findings align with prior studies demonstrating that IL-7 is essential for ILC2 development and persistence (2, 17), and that JAK/STAT pathway components, including JAK3, support ILC2 survival and effector functions (18, 19). Together, our transcriptomic and phenotypic data indicate that RAG1 deficiency enhances signaling networks that promote dILC2 proliferation while also predisposing cells to apoptotic stress. In addition to activation- and cytokine-signaling-associated transcripts, *Rag1^-^/^-^* dILC2s also showed increased expression of selected tissue-homeostasis- or repair-associated transcripts, including Areg, Il9 and Tgfb1. This supports a mixed transcriptional state rather than a purely pro-inflammatory activation program.

Our dataset also revealed increased gene expression of ILC1- and ILC3-associated cytokines, including *Il17a, Il17f, Il22*, and *Ifng*. Such functional transitions have been described in mucosal and inflammatory environments (20) and can be driven by IL-23 and IL-1β, which promote T-bet expression and facilitate ILC2-to-ILC1 conversion while preserving certain type 2 effector functions (20). Elevated transcriptional signatures for IL-23 signaling in *Rag1*^-^/^-^ dILC2s are consistent with these prior observations (14). Although we did not perform protein-level cytokine profiling, these transcriptomic shifts support the concept that dILC2s in *Rag1*^-^/^-^ mice may adopt broader effector programs, possibly compensating for the absence of adaptive immunity.

To assess functional consequences of these RAG1-deficiency-associated differences, we examined responses to DNFB-induced CHS, a robust model of allergic contact dermatitis (21). While dILC2 depletion has been shown to exacerbate CHS in TNCB models (6), the role of dILC2s in DNFB-driven inflammation was unknown. *Rag1*^-^/^-^ mice exhibited significantly reduced ear swelling compared with WT mice, despite their higher steady-state dILC2 numbers. Importantly, WT mice expanded their dILC2 population after DNFB challenge, whereas *Rag1*^-^/^-^ mice showed a reduction in dILC2 abundance. Given that CHS responses depend on both innate and adaptive immunity (21–23), and that DNFB also induces irritant inflammation (21, 22), the attenuated phenotype observed in *Rag1*^-^/^-^ mice likely reflects a combination of impaired adaptive responses and altered dILC2 stress tolerance. The precise mechanisms linking the altered *Rag1^-^/^-^* dILC2 state to the attenuated CHS phenotype remain unresolved. Increased expression of type 2 cytokine genes such as Il13 may contribute to this altered tissue response, as IL-13 has been shown to reduce tissue damage and restrain inflammation in certain contexts, including some cutaneous inflammation models (24).

Adoptive transfer experiments provided a short-term test of whether sensitized WT lymphocytes can modulate *Rag1^-^/^-^* dILC2 responses during the elicitation phase. Short-term adoptive transfer of lymphocytes from DNFB-sensitized donors exerted only a modest, non-significant effect on absolute dILC2 numbers during the elicitation phase. IL-2, produced by adaptive lymphocytes and known to enhance ILC2 proliferation and effector function (5), may have contributed to the trend toward higher dILC2 numbers after transfer. However, the failure of short-term adoptive transfer to restore the WT phenotype suggests that the altered Rag1-deficient host context cannot be fully reversed during the elicitation phase alone and that longer-term immune reconstitution or developmental factors may contribute to the observed dILC2 phenotype.

Mechanistically, *Rag1*^-^/^-^ dILC2s displayed features consistent with altered activation thresholds and impaired stress resilience. Following DNFB challenge and ex vivo stimulation, *Rag1*^-^/^-^ dILC2s predominantly exhibited a CD44^hi^ phenotype, reminiscent of previously described inflammatory ILC2 subsets (10). These cells also demonstrated significantly higher baseline apoptosis and elevated caspase-3/7 activity, suggesting that loss of RAG1 increases their susceptibility to cell death under steady-state conditions. At the same time, *Rag1*^-^/^-^ dILC2s incorporated more BrdU *in vivo* than WT dILC2s, indicating enhanced proliferative activity. The simultaneous increase in proliferation and apoptosis suggests accelerated or dysregulated turnover and an unstable homeostatic equilibrium, which may explain why *Rag1^-^/^-^* mice show expanded dILC2 numbers at baseline but fail to mount a WT-like increase in absolute dILC2 abundance during CHS. Although our data demonstrate stable transcriptional and functional alterations of dILC2s in Rag1-deficient mice, we cannot fully disentangle cell-intrinsic effects from long-term imprinting by the lymphopenic tissue microenvironment. While the adoptive transfer experiments suggest that selected aspects of the phenotype can be modulated during the elicitation phase, incomplete immune reconstitution and altered cytokine milieus in Rag1-deficient hosts may still contribute to the observed effects. Thus, our findings are consistent with durable alteration of the dILC2 compartment in lymphopenic conditions rather than definitive proof of strict cell-autonomous regulation.

Several limitations should be considered. Cell sorting can affect gene expression (25), although WT and Rag1^-^/^-^ samples were processed identically, minimizing genotype-specific bias. Because transcriptomic analyses were performed on sorted dILC2 populations, residual heterogeneity or minor contamination by rare cutaneous lymphoid populations cannot be fully excluded. Transcriptomic analysis required pooling of rare sorted dILC2s to obtain sufficient cell numbers and was performed with limited biological replication. Therefore, the breadth of transcriptional remodeling should be interpreted as hypothesis-generating and was supported by selected protein-level validation where possible. Not all transcriptomic differences were reflected at the protein level, underscoring the need for functional validation. In addition, adoptive transfer experiments did not resolve which lymphocyte subsets modulate dILC2 responses. Moreover, the adoptive transfer experiment did not include a CD3-specific recipient-side engraftment control. Because CD2 and CD3 were acquired in a shared fluorescence channel, the retrospective CD2/CD3 analysis cannot distinguish transferred CD3^+^ T cells from endogenous CD2^+^ innate lymphoid or NK-lineage cells. Accordingly, the present findings are hypothesis-generating and warrant further mechanistic studies. These data suggest that *Rag1^-^/^-^* mice should not be considered immunologically neutral hosts for studies of dermal ILC2 biology, because the dILC2 compartment itself is altered in Rag1-deficient, lymphopenic conditions.

In conclusion, Rag1 deficiency is associated with a distinct dermal ILC2 state characterized by durable transcriptional and functional alteration, abnormal dILC2 dynamics during contact hypersensitivity, and attenuated cutaneous inflammation. These findings indicate that skin inflammation phenotypes in Rag1-deficient mice should be interpreted in light of an altered dermal ILC2 compartment.

## Data Availability

The datasets presented in this study can be found in online repositories. The names of the repository/repositories and accession number(s) can be found below: https://www.ncbi.nlm.nih.gov/, GSE317014<.
