# Supplementary material for: RAG1 deficiency durably alters dermal group 2 innate lymphoid cells and modifies contact hypersensitivity
Source: Front Immunol. 2026 Jul 20;17:1859649. doi: 10.3389/fimmu.2026.1859649 (PMC13431262; doi:10.3389/fimmu.2026.1859649)
Supplement: Supplementary file 1 [file DataSheet1.pdf]

Supplementary Figure 1

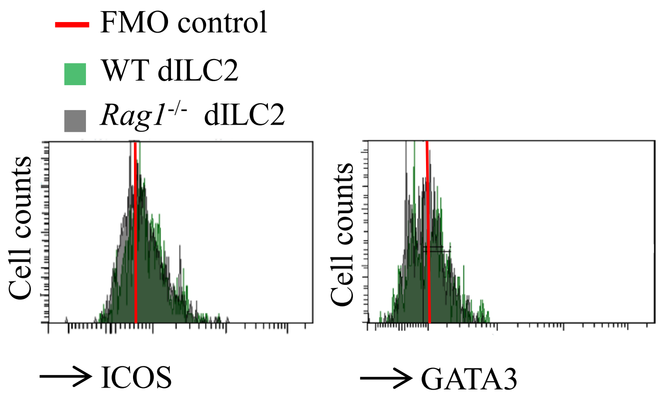

The expression of ICOS and GATA3 in dILC2 of WT and *Rag1*$^{-/-}$ was investigated by flow cytometry. Red line indicates cut-off of FMO control.

Supplementary Figure 2

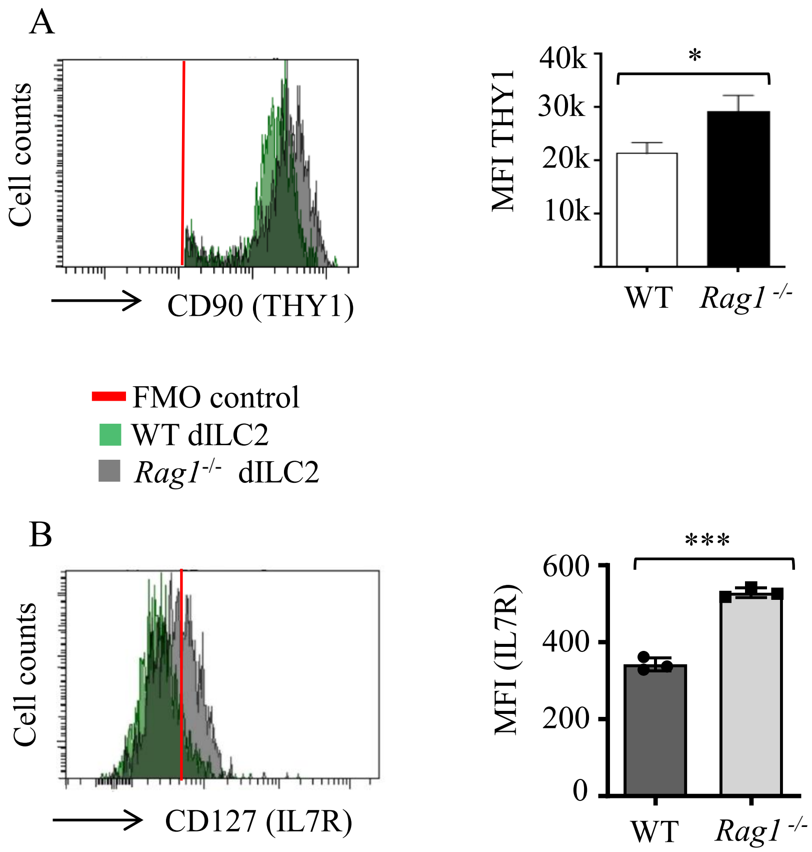

The expression of THY1 (CD90) (A) and IL7R (CD127) (B) in the dILC2 of *Rag1*$^{-/-}$ and WT mice was investigated by flow cytometry (right panels). Red line indicates cut-off of FMO control. Panel A and B represent mean ± SEM (n=3).

Supplementary Figure 3

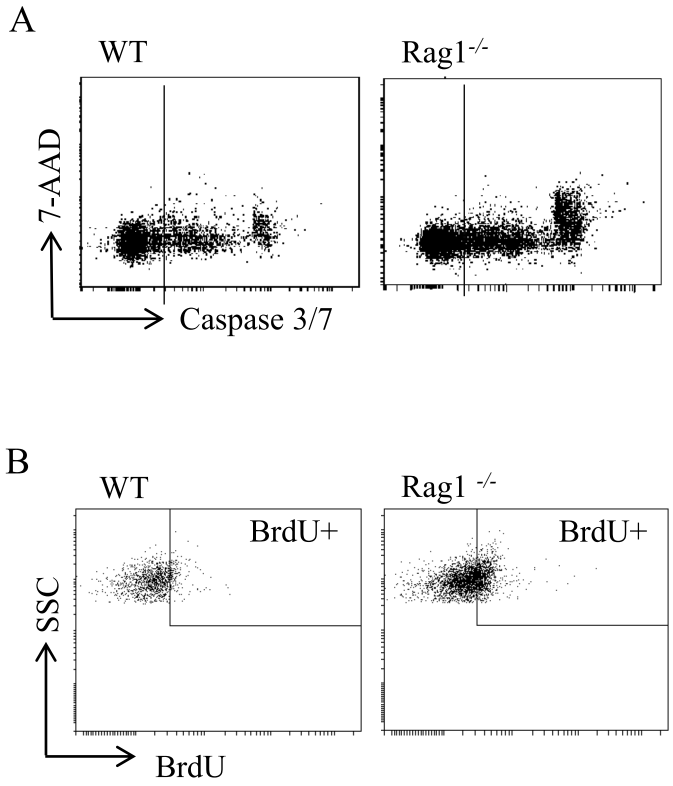

Representative flow cytometry dot plots show caspase 3/7 cleavage (A) and BrdU incorporation (B) of dILC2s from WT and *Rag1*$^{-/-}$.
